# Determination of intramedullary nail based on centerline adaptive registration

**DOI:** 10.3389/fbioe.2023.1115473

**Published:** 2023-03-31

**Authors:** Xiaolong Liu, Jin Li, Kuan Luan

**Affiliations:** College of Intelligent Systems Science and Engineering, Harbin Engineering University, Harbin, Heilongjiang, China

**Keywords:** intramedullary nail, entry point, centerline, femoral shaft fracture, registration

## Abstract

**Objective:** Internal fixation with intramedullary nails is a gold standard for the treatment of femoral shaft fractures. However, both the mismatch between intramedullary nails and the medullary cavity and inaccurate positioning of entry points will lead to deformation of intramedullary nails after implantation. The study aimed to determine a suitable intramedullary nail with an optimal entry point for a specific patient based on centerline adaptive registration.

**Method:** A homotopic thinning algorithm is employed to extract centerlines of the femoral medullary cavity and the intramedullary nail. The two centerlines are registered to obtain a transformation. The medullary cavity and the intramedullary nail are registered based on the transformation. Next, a plane projection method is employed to calculate the surface points of the intramedullary nail laid outside the medullary cavity. According to the distribution of compenetration points, an iterative adaptive registration strategy is designed to decide an optimal position of the intramedullary nail in medullary cavity. The isthmus centerline is extended to the femur surface, where the entry point of the intramedullary nail is located. The suitability of an intramedullary nail for a specific patient was calculated by measuring the geometric quantities reflecting the interference between the femur and nail, and the suitability values of all nails are compared and the most suitable one is determined.

**Results:** The growth experiment indicated that the bone to nail alignment is indeed affected by the extension of the isthmus centerline, including the extension direction and velocity. The geometrical experiment showed that this method could find the best registration position of intramedullary nails and select the optimal intramedullary nail for a specific patient. In the model experiments, the determined intramedullary nail could be successfully placed into the medullary cavity through the optimal entry point. A pre-screening tool to determine nails which can be successfully used has been given. In addition, the distal hole was accurately located within 14.28 s.

**Conclusion:** These results suggest that the proposed method can select a suitable intramedullary nail with an optimal entry point. The position of the intramedullary nail can be determined in the medullary cavity, while deformation is avoided. The proposed method can determine the largest diameter intramedullary nail with as little damage to the intramedullary tissue as possible. The proposed method provides preparation aid for internal fixation with intramedullary nails guided by navigation systems or extracorporeal aimers.

## 1 Introduction

Femoral shaft fractures account for about 6% of systemic fractures (Saleeb et al., 2019). Most of femoral shaft fractures are caused by violent shocks and occur in young adults. At present, intramedullary nail (IMN) internal fixation and steel plate internal fixation are two common minimally invasive surgeries for the clinical treatment of femoral shaft fractures. The successful healing of fractures depends on early moderate mechanical load (Bailon-Plaza and van der Meulen, 2003). Plate internal fixation usually cannot provide enough support for early rehabilitation (Liu et al., 2019). In addition, the Marchetti-Vicenzi’s nail has been proved to treat humerus diaphyseal fractures, and some very meaningful studies are aimed to optimize this device to treat femoral fractures (Pascoletti et al., 2018). Up to now, IMN internal fixation is still the routine treatment of femoral shaft fracture (Gao et al., 2021). Distal locking has become the biggest challenge in surgery, accounting for an average of 1/3 of the operation time (Ioannis et al., 2013). In addition, X-ray is used most frequently, causing a lot of radiation damage to medical staff and patients (Ma et al., 2018). The difficulty of distal locking with an extracorporeal aimer is mainly due to the deformation of IMNs after insertion (Diotte et al., 2015). The three main factors leading to the deformation include: position deviation of the entry point (Prasarn et al., 2010), poor matching between IMNs and the medullary cavity (MC) (Liaw et al., 2019), and incorrect placement of IMNs. In clinical practice, determining IMN mostly depends on the personal experience of surgeons. The incorrect selection of IMN may lead to poor surgical efficacy and decrease the probability of fracture healing. If the surgery fails, it may seriously affect the patient’s health.

The application of intelligent methods of computer science and digital technology brings new possibilities to solve the deformation of IMNs after insertion. The development of multibody models provides a numerical tool for the pre-surgical screening of internal fixation devices and foresees the nails’ performance before clinical applications (Putame et al., 2020). Mortazavi et al. (2018) predicted postoperative nail deformation by establishing a finite element model of the femur of a specific patient and simulating the mechanical interaction between the femur and the nail during nail insertion. Then they proposed a method to predict nail deformation during surgery by using orthogonal preoperative X-ray images (Mortazavi et al., 2019). Esfandiari et al. (2016) extracted the features of interest from a sequential biplanar set of X-ray imagery of the distal part of an inserted IMN and calculated the six degree-of-freedom position and orientation parameters of the nail. Some researchers investigate this problem from the perspective of measuring the morphological characteristics of MCs. For example, Liaw et al. (2019) proposed a region growth algorithm to obtain the radiuses of anterior femoral arches. The radiuses can be used to guide the design of IMNs. de Almeida et al. (2017) presented an automated workflow to extract femoral orientation and landmarks such as the femoral neck axis and the femoral middle diaphysis axis. According to the extracted parameters, the appropriate prosthesis and placement positions can be selected for a specific patient. Buford et al. (2014) measured the geometry and size of the lateral and medial anterior cortices of the femur and associated the resulting curvature of the femur with the curvature of existing IMNs. The relation can be used to guide the selection of a suitable IMN. Pascoletti et al. (2021) employed principal components analysis to extract the principal components of proximal femurs, through which the three-dimensional (3D) design of an IMN can be easily parametrized to cover the specific patient’s need. Su et al. (2015) measured the anatomical parameters of femurs and isthmuses using a geometric analysis software and proposed a new method for measuring the MC and predicting the implant’s size; Chen et al. (2018) employed a k-means clustering method of density peak to divide 422 femurs into three different subtypes according to the quantitative morphological characteristics of MCs. This classification can guide the design of unique IMN. Inaccurate positions of entry points can also lead to the deformation of IMNs (Prasarn et al., 2010). To choose an optimal insertion position, Farhang et al. (2014) dissected pairs of femurs of 374 cadavers and measured the offset of the apex of the greater trochanter relative to the femoral axis. Finally, they concluded that the insertion position should be about 5 mm behind the apex of the greater trochanter. Zhao et al. (2015) developed a new method for measuring the anatomical structure of the proximal femur and locating the optimal entry point of IMN. The anatomical parameters of 200 Chinese femurs were measured and analyzed, and the most relevant factors for the best insertion point were determined. However, these two methods only predict the range of insertion positions and do not locate the optimal entry point for a specific IMN. Byun and Jung (2016) simulated the insertion of an anterograde IMN into the 3D model of the femur in Mimics. The relationship between the ideal entry point and the morphological characteristics of femoral neck was analyzed. However, the simulated insertion still depends on the subjective judgment of doctors which may cause human errors. Oszwald et al. (2010) developed an entry point drilling robot, which used two orthogonal fluoroscopic images of the proximal femur to automatically calculate the femoral axis. The entry point was located on the extension of this axis. Compared with the manual technique, this method reduces the number of fluoroscopic images required from 11.6 to 4 during drilling. However, this method is not applicable for proximal lateral bending IMNs.

Both an unsuitable IMN and inaccurate positioning of an entry point will lead to deformation of the IMN after implantation. The above studies only solve one of the two problems. If either of them is not solved, the IMN may still deform. One possible solution is to avoid the mismatch and fault location at the same time. This study will try to solve these two problems at the same time from a new perspective.

## 2 Methods

### 2.1 Overview of methods

**FIGURE 1:**
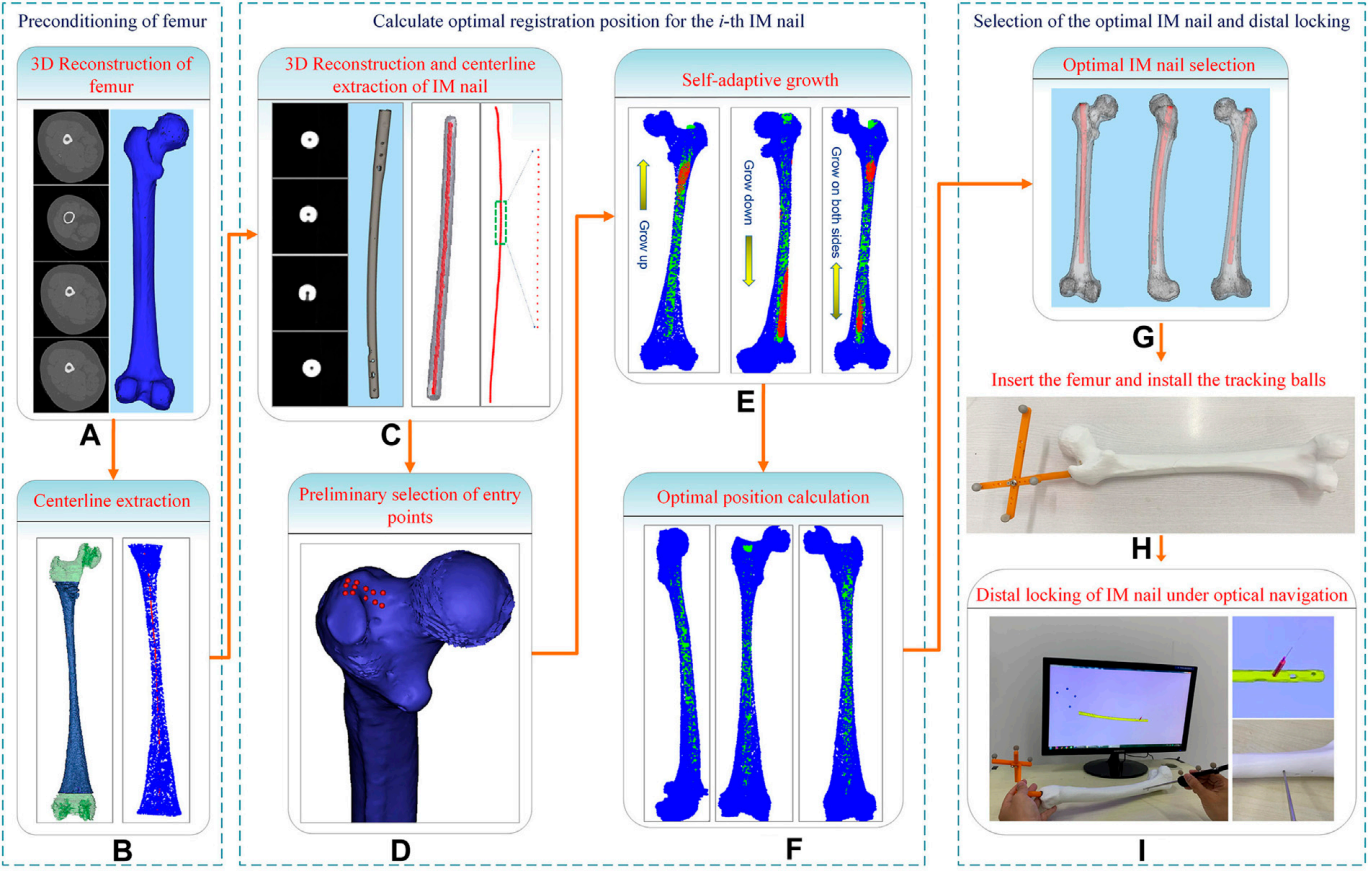
System flow chart of the computer-assisted preoperative selection method.

In this study, a computerized preoperative selection method is proposed for the treatment of femoral fractures with intramedullary nailing. The processing flow is as follows (Figure 1): First, the CT images of a femur are segmented and a 3D model is reconstructed. A centerline is extracted from the interested region of the MC. Second, the CT images of an IMN are segmented and its 3D model is reconstructed. The whole centerline of the IMN is extracted. Third, an entry point is preliminarily selected. The path from the entry point to the end of MC centerline is determined. Fourth, the centerline of the MC isthmus and the centerline of the IMN are registered. An optimal registration scheme is established to reduce the IMN surface points located outside the MC. Fifth, the IMN is registered to the MC based on the transformation obtained from the fourth step. Sixth, the operations of steps 2 to 5 are performed on each IMN. A suitable IMN can be determined with optimal orientation or alignment and entry point. Seventh, the distal nail hole can be locked accurately guided by a navigation system.

### 2.2 Centerlines extraction of IMN and MC

**FIGURE 2:**
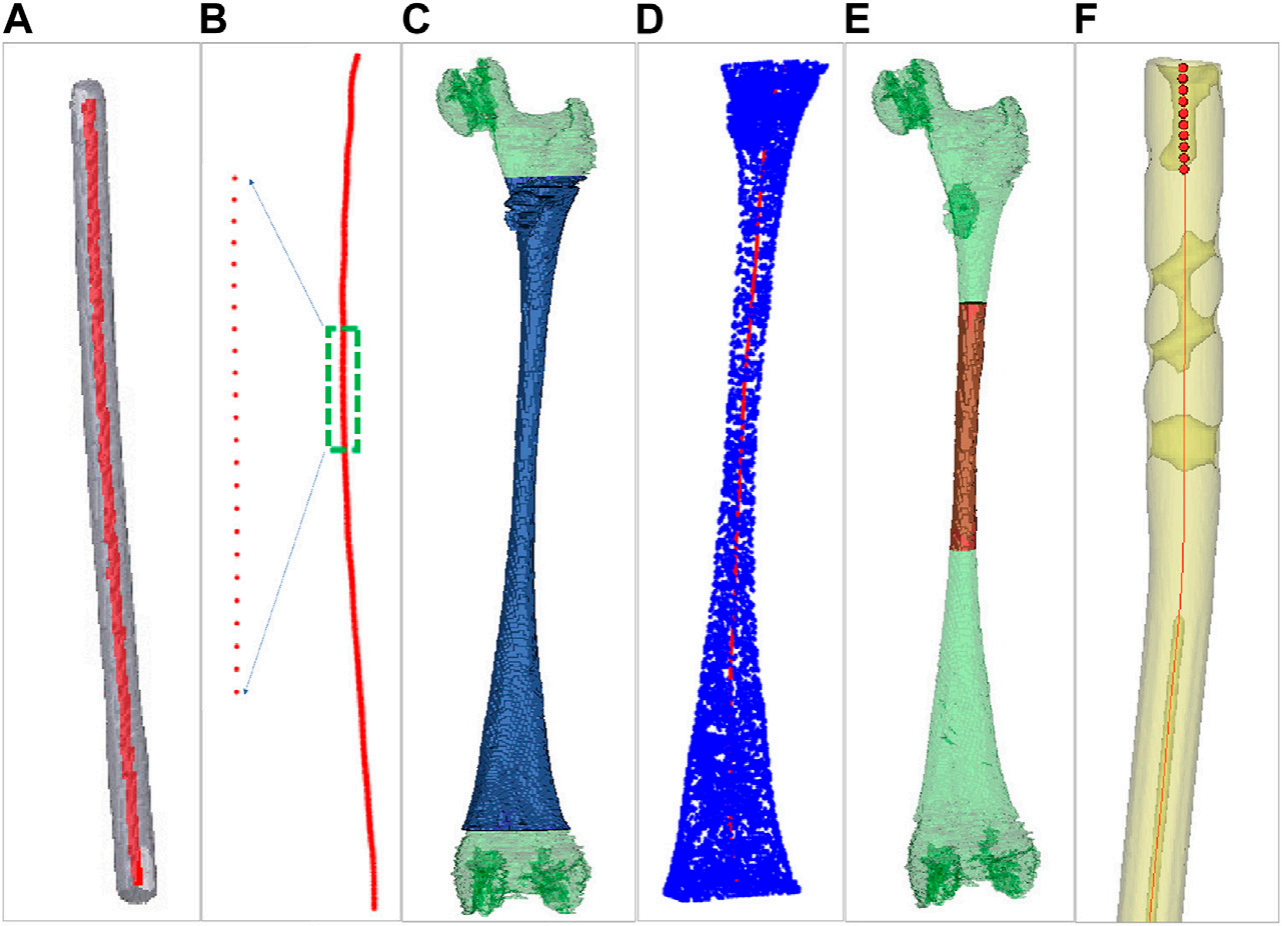
Centerlines extraction of the intramedullary nail and the medullary cavity. **(A)** Centerline extraction of the intramedullary nail. **(B)** Setting control points. **(C)** Green for the medullary cavity and blue for the interested part. **(D)** Centerline extraction of the interested part. **(E)** Green for the medullary cavity and red for the medullary cavity isthmus. **(F)** The ten continuous control points near the upper end of intramedullary nail centerline.

For both men and women, the left and right lower limbs maintain almost the same length, which is beneficial to maintain the balance of human bodies, and there is no significant difference between the left and right axes of the lower limbs under CT examination (Ferras-Tarrago et al., 2020). To facilitate the extraction of a femur centerline, images of the healthy side bone are used in this study. The healthy side bone and a candidate IMN are imaged by a CT scanner, and the DICOM sequence images are obtained. The Marching Cubes algorithm (Masala et al., 2013) is employed to reconstruct surface models of the femur (Figure 1A) and the IMN (Figure 1C) in three dimensions. The IMN is processed first. The centerline of the IMN is extracted by a homotopic thinning algorithm (Kerschnitzki et al., 2013). As a result, a central axis with a single voxel thickness is obtained, which is homotopic with the original image (Figure 2A). Then, the central axis is fitted as a curve line. A serial of control points with 1 mm interval are located on the curve line. For each control point, a fitting circle of IMN cross-section is placed. The diameter of the fitting circle and the curvature of IMN centerline around the control point are also recorded (Figure 2B).

Next, a 3D model of the healthy femur is imported into a 3D model processing software (Geomagic Control 2014). All the surface points of femur are removed manually, and the 3D model of the MC remains. Since the proximal end of femur includes the femoral head and the greater trochanter, and the distal end includes the condylar bone. The irregular shapes of both ends of the femur will affect the accuracy of extracting MC centerline. We only extract the middle tubular part as the interested part (Figure 2C). The centerline of the interested part is also extracted by the same homotopic thinning algorithm and control points are produced. The diameter of the fitting circle on the cross-section of the MC at each control point and the curvature of the MC centerline here are both recorded (Figure 2D).

### 2.3 Preliminary selection of entry point based on the centerline of MC

Since the diameters of the distal part of an IMN are almost the same, while the MC is thick at both ends and thin in the middle. Besides, the curvature changes obviously in the middle narrow part of MC (marked as the MC isthmus) (Figure 2E). Therefore, the registration between the MC isthmus and the IMN is the key. Only the centerline of the interested part of MC is extracted in the previous step, so there is still a gap between the extracted centerline end and the apex of the greater trochanter. In order to locate an entry point and fill the gap, the intersection angle between the femoral neck’s axis and the shaft’s axis in 3D space, the femoral radius, the head radius, and the femoral length were measured. According to the method in Zhao et al. (2015) (Figure 1D), a possible area of entry points was determined and we manually select twelve candidate entry points. An interpolation method is used to place the control points between the candidate entry points and the starting point of interested part centerline respectively. Twelve central axes from the candidate entry points to the endpoint of interested part centerline which is near the intercondylar fossa are obtained. Next, we will find the most suitable one from the 12 central axes. If the control points on the one of central axes are used as the target point set and the control points on the IMN centerline as the source point set, a registration based on Iterative Closest Point (ICP) algorithm can be performed. Because ICP is easy to fall into the local optimal solution (Sobreira et al., 2019), this study combines Normal Distributions Transform (NDT) and ICP algorithm for registration (Shi et al., 2018). After registration, three influencing factors are calculated: curvature difference, diameter difference, and distance difference. The curvature difference between *jth* (*j* = 1, 2, … 12) central axis and the IMN centerline is calculated as Eq. 1:

$$C_{j}=\sum_{i=1}^{k}\left.{\left|C\right.}_{nail}^{i}-C_{cavity}^{i}\right|$$ (1)

where *k* is the number of control points of the IMN centerline, $C_{nail}^{i}$ and $C_{cavity}^{i}$ are curvature at *ith* control points of the IMN centerline and the MC centerline. $C^{\mathrm{Max}}$ and $C^{\mathrm{Min}}$ denote the maximum value and minimum value of the set $\left\{C_{1},C_{2},\ldots C_{12}\right\}$ respectively. The normalization $C_{j}^{M}$ of $C_{j}$ is processed as follows:

$$C_{j}^{M}=\frac{C_{j}-C^{\mathrm{Min}}}{C^{\mathrm{Max}}-C^{\mathrm{Min}}}$$ (2)

The same operation in the calculation of $C_{j}^{M}$ is performed on the diameter difference to obtain $D_{j}^{M}$ . $D_{j}^{M}$ represents the normalization of the diameter difference between *jth* central axis and the IMN centerline. The distance difference between *jth* central axis and the IMN centerline is calculated as Eq. 3:

$$E_{j}=\sum_{i=1}^{k}E^{i}$$ (3)

where $E^{i}$ is the Euclidean distance between *ith* control point of the IMN centerline and *i*th control point of the MC centerline. The normalization $E_{j}^{M}$ of $E_{j}$ is calculated using the method in Eq. 2. The fitness of *jth* central axis registered with the IMN centerline is calculated as Eq. 4:

$$\varphi_{j}=\frac{C_{j}^{M}}{3}+\frac{D_{j}^{M}}{3}+\frac{E_{j}^{M}}{3}$$ (4)

The fitness *φ* varies between 0 and 1. The smaller the *φ* is, the more appropriate the central axis fits the IMN centerline. The most appropriate central axis is found out, and its corresponding entry point is used as the initial entry point.

### 2.4 Adaptive growth of the centerline of MC isthmus

**FIGURE 3:**
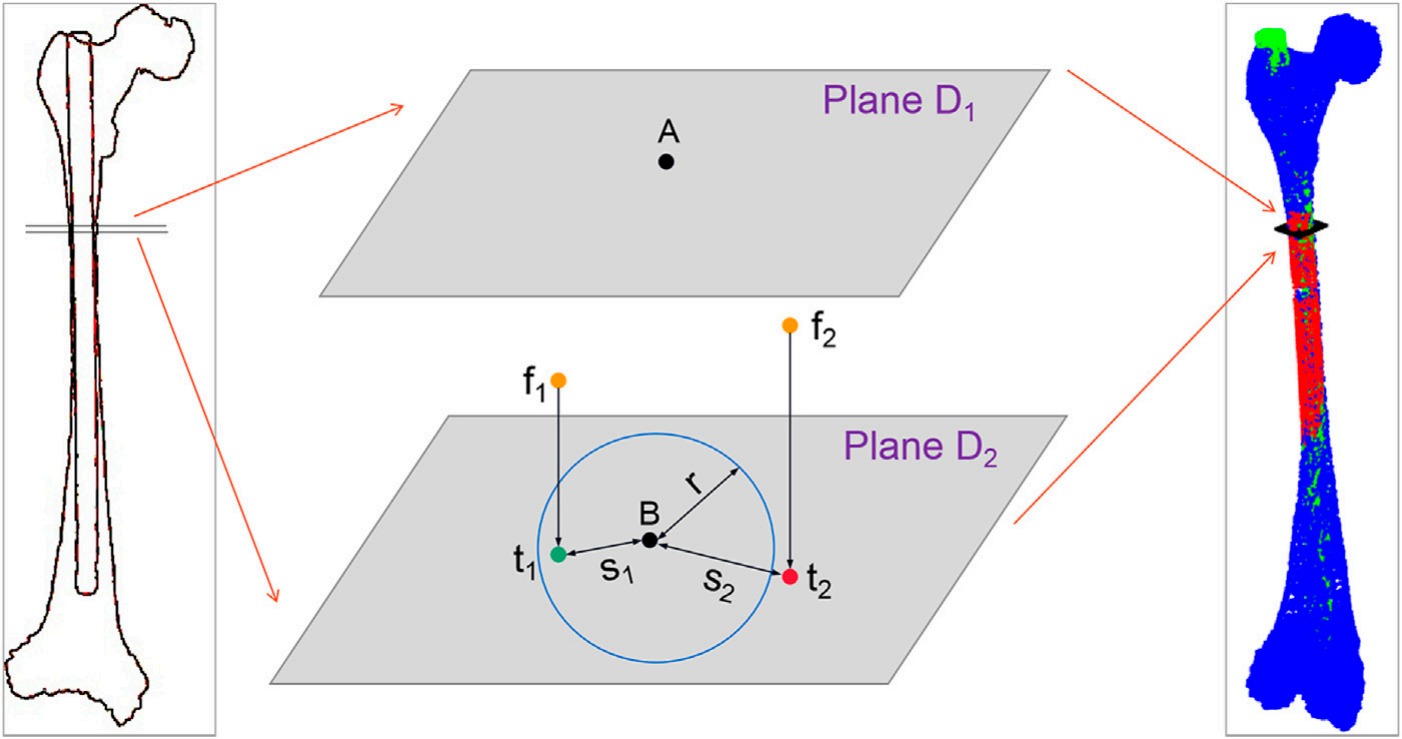
Schematic diagram of outside-points calculation. Where *f*_*1*_ and *f*_*2*_ are the two points in the point set *P*_*middle*_, and *t*_*1*_ and *t*_*2*_ are their projection points. *s*_*1*_ and *s*_*2*_ are the distances from the two projection points to control point *B*, and the blue circle represents the fitting circle of the medullary cavity cross-section at control point (B).

In this part, a plane projection method is employed to calculate the surface points of the IMN laid outside the MC. According to the distribution of compenetration points, an iterative optimization of nail orientation, based on more and more extended volume regions including the isthmus is performed. Firstly, the narrowest part of the MC is calculated, and the initial length is set to 90 mm, which is used as the MC isthmus. Then, the centerline of the MC isthmus is intercepted. A centerline with the same length at the corresponding position of IMN is also intercepted. The two centerlines are registered by NDT and ICP algorithm, and a spatial transformation matrix *T* is obtained. Based on the transformation matrix *T*, the IMN is registered with the MC. We use the number of surface points of the IMN located outside MC to optimize the registration. If a point on the IMN falls outside the MC, it means that the IMN squeezes the inner wall of the femur there. Here are the processing steps:

(1) Acquisition of the surface points of the IMN falling between the adjacent control points of the MC centerline: As shown in Figure 3, *A* and *B* denote two adjacent control points on the MC centerline. Plane *D*_*1*_ and plane *D*_*2*_ are cross sections that pass through point *A* and point *B* respectively and are perpendicular to the line connecting points *A* and *B.* All the points between planes *D*_*1*_ and *D*_*2*_ in the transformed IMN point cloud are saved in point set *P*_*middle*_ *.*
(2) Search of the outside point and calculation of the exceeded distance: Point *f* is a point in the point set *P*_*middle*_ *.* Point *f* is projected to plane *D*_*2*_ to get the projection point *t.* The distance (marked as *s*) from *t* to control point *B* is calculated and compared with the MC radius (marked as *r*) at control point *B.* If *s* ≤ *r*, it means that point *f* falls inside the MC. If *s* > *r*, point *f* is located outside the MC, and point *f* is called the outside point and is saved in the point set *P*_*out*_ *.* $d_{f}=s-r$ represents the exceeded distances of point *f.*
(3) Calculation of the number of outside points and the mean exceeded distance: Step 2) is performed on all the points in *P*_*middle*_ *.* All the points located outside the MC in *P*_*middle*_ are found and saved in the point set *P*_*out*_ *.* Step 1) is performed on all the adjacent control points on the MC centerline in turn, and *P*_*middle*_ is updated constantly with each operation. All the points in the updated *P*_*middle*_ are operated in step 2), and the points located outside the MC are saved in *P*_*out*_ *.* Finally, all the points located outside the MC on the whole IMN are saved in *P*_*out*_ *.* The number of outside points is marked as Noutp . At the same time, it is also necessary to calculate the mean exceeded distance. The mean exceeded distance (marked as Doutp ) is the sum of the exceeded distances of all outside points divided by Noutp *.*

**FIGURE 4:**
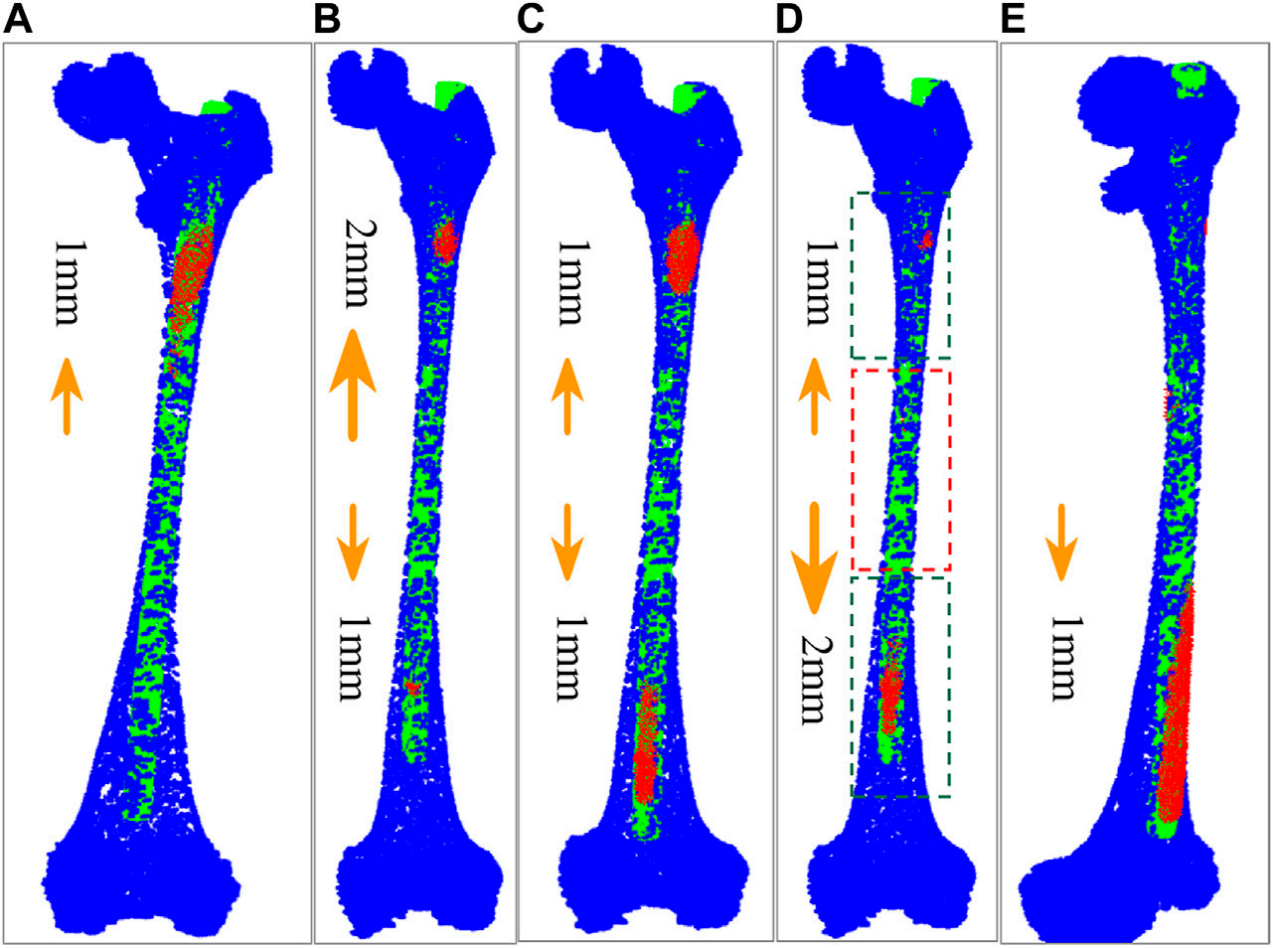
Five growth schemes determined according to the distribution of outside points. **(A)** The centerline of MC isthmus increases 1 mm upward at a time. **(B)** The isthmus centerline increases 2 mm upward and 1 mm downward at a time. **(C)** The isthmus centerline increases 1 mm upward and downward respectively each time. **(D)** The isthmus centerline increases 1 mm upward and 2 mm downward each time. The part in red dashed contour is the isthmus, while the parts in green dashed contours represent the growing volumes. **(E)** The isthmus centerline increases 1 mm downward each time.

We find that some points fall outside the MC after calculation because the isthmus length is too short to take into account the registration of both ends. The best registration position can be found by changing the length of the isthmus centerline and registering many times to reduce the number of outside points. However, there are many options for the growth direction and length of the isthmus centerline. If all the options are performed separately, the calculation efficiency will be greatly reduced. Therefore, we propose an iterative adaptive registration method to quickly find the effective length and position of the isthmus centerline. In this method, the isthmus centerline can determine the growth direction and growth speed autonomously according to the distribution of outside points. The MC is divided into upper and lower parts bounded by the midpoint of the isthmus centerline. We assume that the number of totally outside points of the IMN is Noutp , and the number of points beyond the upper MC is Noutup . If Noutp ≥ Noutup > 4/5 Noutp , the centerline of the MC isthmus increases 1 mm upward at a time (Figure 4A). If 4/5 Noutp ≥ Noutup > 3/5 Noutp , the isthmus centerline increases 2 mm upward and 1 mm downward at a time (Figure 4B). If 3/5 Noutp ≥ Noutup > 2/5 Noutp , the isthmus centerline increases 1 mm upward and downward respectively each time (Figure 4C). The isthmus centerline increases 1 mm upward and 2 mm downward if 2/5 Noutp ≥ Noutup > 1/5 Noutp (Figure 4D), and 1 mm downward only every time if 1/5 Noutp ≥ Noutup ≥ 0 (Figure 4E). The growing will stop when the MC isthmus centerline grows to the endpoint. The growth direction and growth speed are determined according to the proportion of Noutup to Noutp . The proportion is obtained by a large number of experiments. We adopt the corresponding growth scheme when Noutup is in a certain range, and the IMN and MC can quickly achieve the best registration effect.

### 2.5 Calculation of the optimal placement position and entry point

When the isthmus centerline increases, the centerline with the same length at the corresponding position of IMN also increases. According to the determined growth scheme, each increase of the isthmus centerline is re-registered with the IMN centerline to obtain a new transformation matrix *T*. The IMN is registered with the MC based on *T*. The number of outside points Noutp , the mean exceeded distance Doutp , and the mean distance (marked as Econtrolp ) under the registration are calculated and recorded. The value of Econtrolp is calculated as Eq. 5:

Econtrolp=∑i=1ndi/n (5)

where *n* is the number of control points in the IMN centerline. $d_{i}$ is the Euclidean distance between *ith* control point of the MC centerline and *ith* control point of the IMN centerline. The normalization NoutM of Noutp is processed as follows:

NoutMj=Noutpj−NoutpMinNoutpMax−NoutpMin (6)

where Noutpj is the number of outside points under the registration after *jth* increase. NoutpMax and NoutpMin denote the maximum value and minimum value of all values of Noutp after each increase. The same operation is performed on Doutp and Econtrolp to obtain DoutM and EcontrolM . The fitness of the IMN registered with the MC after *jth* increase is calculated as Eq. 7:

$$\Psi_{j}=\frac{{{}_{out}^{M}N}_{j}}{2}+\frac{{{}_{out}^{M}D}_{j}}{4}+\frac{{{}_{control}^{M}E}_{j}}{4}$$ (7)

**FIGURE 5:**
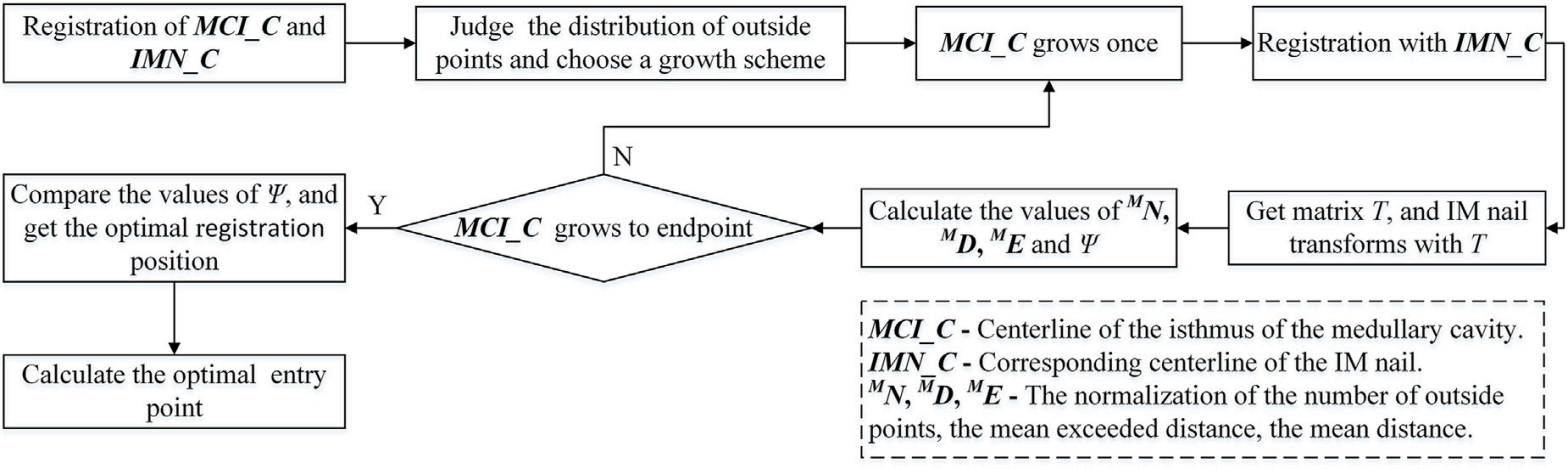
Flowchart of the calculation of the optimal registration position.

The optimal growth times can be found by comparing the values of *Ψ* of each increase. At the same time, we notice that the selection of the initial length of the isthmus centerline will also affect the accuracy of registration. The initial length is set to 70 mm, 80 mm, 100 mm, and 110 mm respectively. The isthmus centerline increases again according to the determined growth scheme and registers with the IMN centerline multiple times. Finally, the optimal growth times and transformation matrix under the optimal isthmus length are obtained. The position of IMN based on the transformation matrix is the optimal registration position (Figure 1F). The main computing process is shown in Figure 5.

The correct location of the entry point is very important for smooth insertion of the IMN and no deformation after insertion. In this study, a new solution is developed to choose an optimal entry point. After the IMN is registered at the optimal position, ten continuous control points are selected near the upper end of the IMN centerline (Figure 2F). The ten control points are fitted as a straight line, and the line is extended to the surface of femoral greater trochanter. Distances between all the proximal femoral surface points and the line are calculated successively. The point with the shortest distance is adopted as the optimal entry point. In clinical practice, the femoral image coordinate system and the patient coordinate system will be registered. Surgical instruments equipped with spatial trackers can locate the actual entry point on the femur according to the determined entry point on the femoral image. Then, the opening operation of the entry point can be guided by spatial navigation systems.

### 2.6 Determination of the optimal IMN

In clinical surgeries, the first thing is to judge whether the length of an IMN is suitable or not. Ideally, the distal end of the IMN should be located between the patella upper edge and the bone scale line. Too long IMN will destroy the cortical bone of intercondylar fossa or hinder incision suture. Too short will affect the stability of fracture fixation and the function of hip joint and knee joint, and may even lead to postoperative re-fracture. In this study, an appropriate length of IMNs is determined between the length of MC centerline −3.5 cm and the length of MC centerline. Similarly, the diameters of IMNs should also be considered. A large diameter will lead to implantation failure, and a small one will lead to insufficient support strength. In this study, the maximum diameter of the lower half of IMN is determined between the minimum cross-section diameter of MC -3 mm and the minimum cross-section diameter of MC. If an IMN does not meet the length and diameter requirements, no further processing will be carried out. IMNs that meet the length and diameter requirements perform steps 1 to 5 of the method in sequence, and the optimal registration positions of all the IMNs are obtained. The number of outside points Noutp , the mean exceeded distance Doutp and the mean distance Econtrolp of each IMN under their corresponding optimal registration are calculated and recorded. The support strength of IMN (marked as SIMNd ) is also an influencing factor that must be considered, which involves the early weight-bearing exercise of patients. The support strength of IMN is mainly related to the anti-torsion strength and the anti-bending strength, which are proportional to the cube of IMN diameter. We use the cube of the difference between the minimum cross-sectional diameter of MC and the maximum diameter of the lower half of IMN to characterize the support strength, and the maximum difference is set to 3 mm. The universal measure NoutUM of Noutp is processed as follows:

NoutUMj=Noutpj−NoutpminNoutpmax−Noutpmin (8)

where Noutpj is the number of outside points of *jth* IMN under its optimal registration with the MC. The minimum of Noutp donated Noutpmin is 0. Noutpmax represents the maximum number of IMN surface points that are allowed to exceed outside the MC. According to the registration results of multiple IMNs and medullary cavities, Noutpmax is 120 which is an empirical value obtained from many tests. Similarly, the registration results also get that the maximum value of Doutp is 0.47 mm, and the maximum value of Econtrolp is 5 mm. Because of the relatively small sample size, all the maximum values are temptative values which require further studies. All the minimum values of Noutp , Doutp , Econtrolp and SIMNd are 0. The same operation in calculation of NoutUM is performed on Doutp , Econtrolp and SIMNd to obtain DoutUM , EcontrolUM and SIMNUM . The suitability *Φ* of *jth* IMN for the MC is calculated as Eq. 9:

Φj=2×NoutUMj5+DoutUMj5+EcontrolUMj5+SIMNUMj5 (9)

The smaller the *Φ* is, the higher matching between the IMN and the MC is. The most suitable IMN for the patient is determined by comparing the values of *Φ* (Figure 1G).

## 3 Experiments and results

**FIGURE 6:**
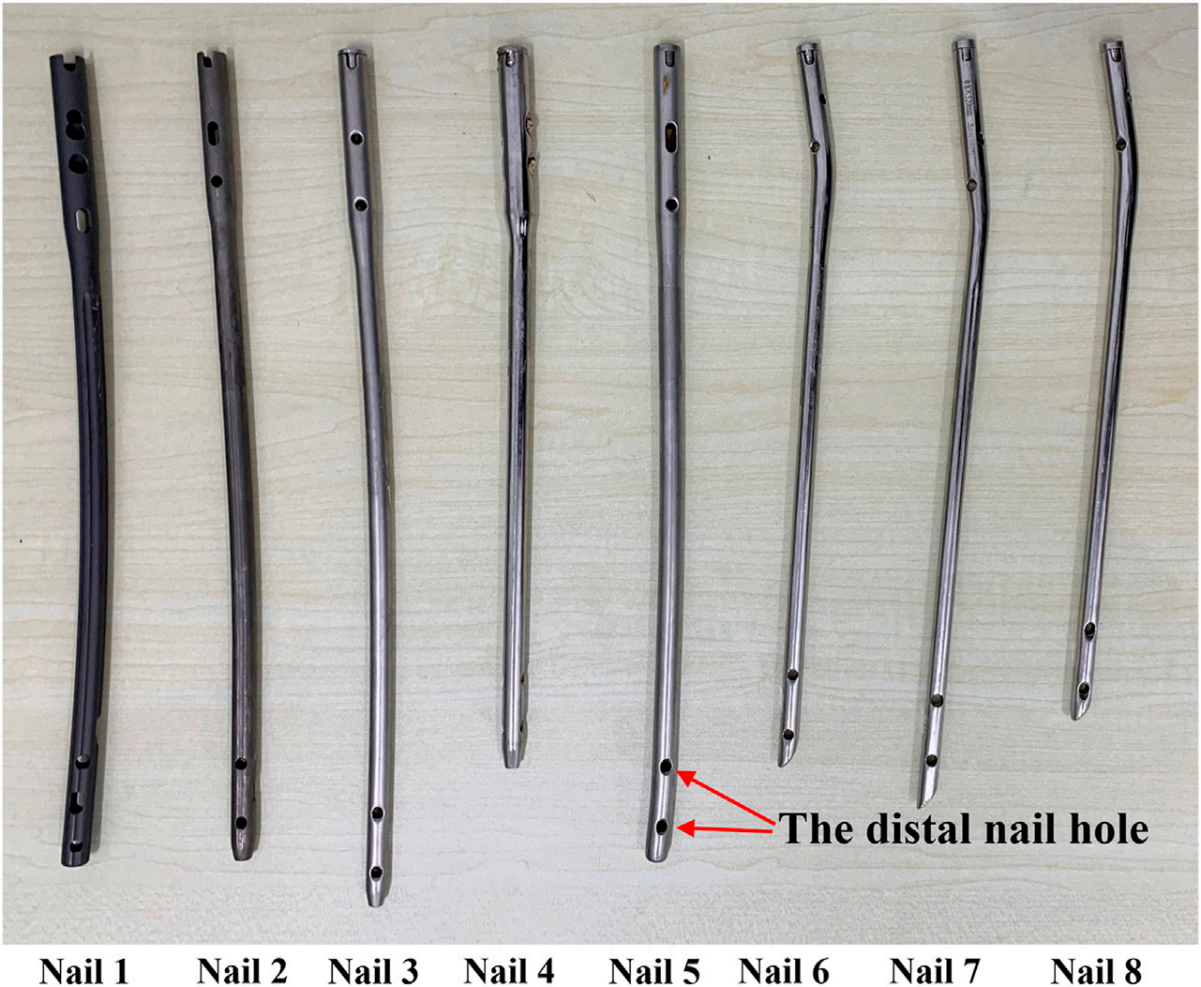
The intramedullary nails used in the experiments are one to eight from left to right.

Four patients’ femurs images and 8 IMNs (Figure 6) were used in the experiments. The femoral images were obtained from the first affiliated Hospital of Harbin Medical University. An optical position sensor (Polaris Vicra, Northern Digital Inc., Canada) and its position probe were employed in experiments to obtain the positions of IMNs and femurs.

### 3.1 Experiment of the centerline growth of the MC isthmus

The effectiveness of the registration between IMNs and MCs based on the centerline growth scheme of MC isthmus was verified in this experiment. Patient 1 was taken as an example. The No. Two IMN was the most suitable IMN for patient 1 determined by the proposed method. The adopted growth scheme of the isthmus centerline was to increase 1 mm upward and 0 downward each time. According to the calculation result, the best registration length was reached in 27 growth times. Five different growth times were selected according to the rule of sequential increase, and there were two growth times before and after the optimal growth times respectively. The five growth times of patient 1 were first, 14th, 27th, 40th and 53rd growth. The five growth times were denoted as times 1, 2, 3, 4 and 5. Similarly, the five growth times of patient 2 were first, 13th, 27th, 39th and 51st growth. The five growth times of patient 3 were first, 13th, 25th, 37th and 49th growth. While patient 4 were first, fifth, eighth, 20th and 35th growth. The values of Noutp , Doutp and Econtrolp of registration between the MC of patient 1 and its optimal IMN at 5 different growth times were calculated and recorded in Table 1. The same operation was performed on the remaining patients respectively, and the values of Noutp , Doutp , and Econtrolp were also recorded in Table 1.

**TABLE 1:** Registration of different growth times of the isthmus centerlines in 4 patients.

		Times 1	Times 2	Times 3	Times 4	Times 5
Patient 1	Noutp	718	281	0	5	27
	Doutp (mm)	1.377	0.609	0	0.059	0.101
	Econtrolp (mm)	3.256	2.939	2.928	2.981	3.030
Patient 2	Noutp	219	85	0	2	5
	Doutp (mm)	0.508	0.261	0	0.047	0.095
	Econtrolp (mm)	1.936	1.890	1.847	1.891	1.923
Patient 3	Noutp	90	2	0	14	32
	Doutp (mm)	0.169	0.018	0	0.076	0.159
	Econtrolp (mm)	1.981	1.554	1.458	1.476	1.487
Patient 4	Noutp	26	5	0	2	4
	Doutp (mm)	0.136	0.045	0	0.033	0.036
	Econtrolp (mm)	1.922	1.859	1.831	2.003	1.198

As shown in Table 1, all Noutp , Doutp and Econtrolp decrease at first and then increase with the increase of growth times, and all get the minimum value at the optimal growth times. Therefore, the registration is affected by the position and length of the isthmus centerline. The result indicates that the proposed method can obtain an appropriate centerline length of the MC isthmus.

### 3.2 Geometrical experiment

**FIGURE 7:**
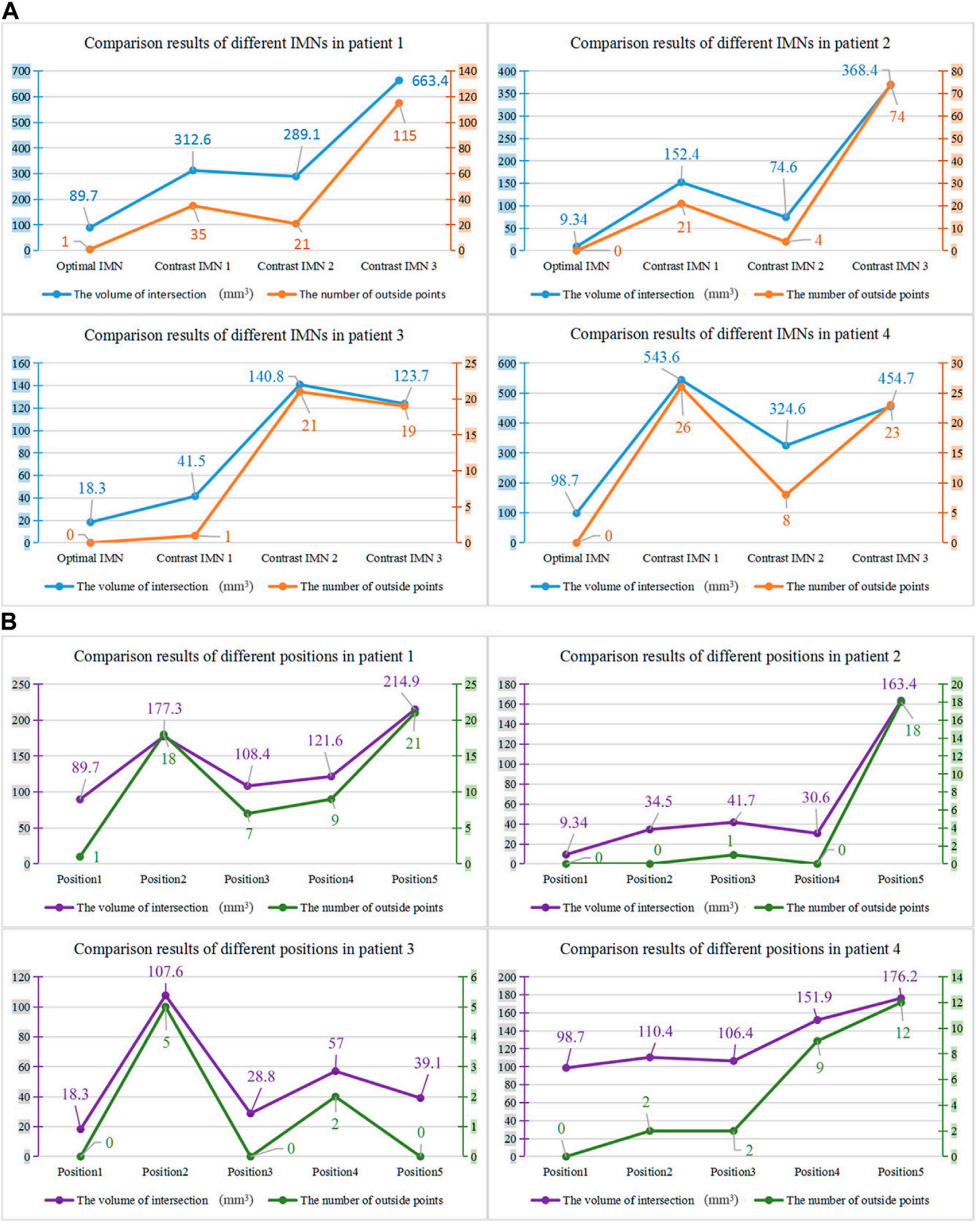
**(A)** Comparison results of different intramedullary nails for the four patients. **(B)** Comparison results of different registration positions of the four patients.

**FIGURE 8:**
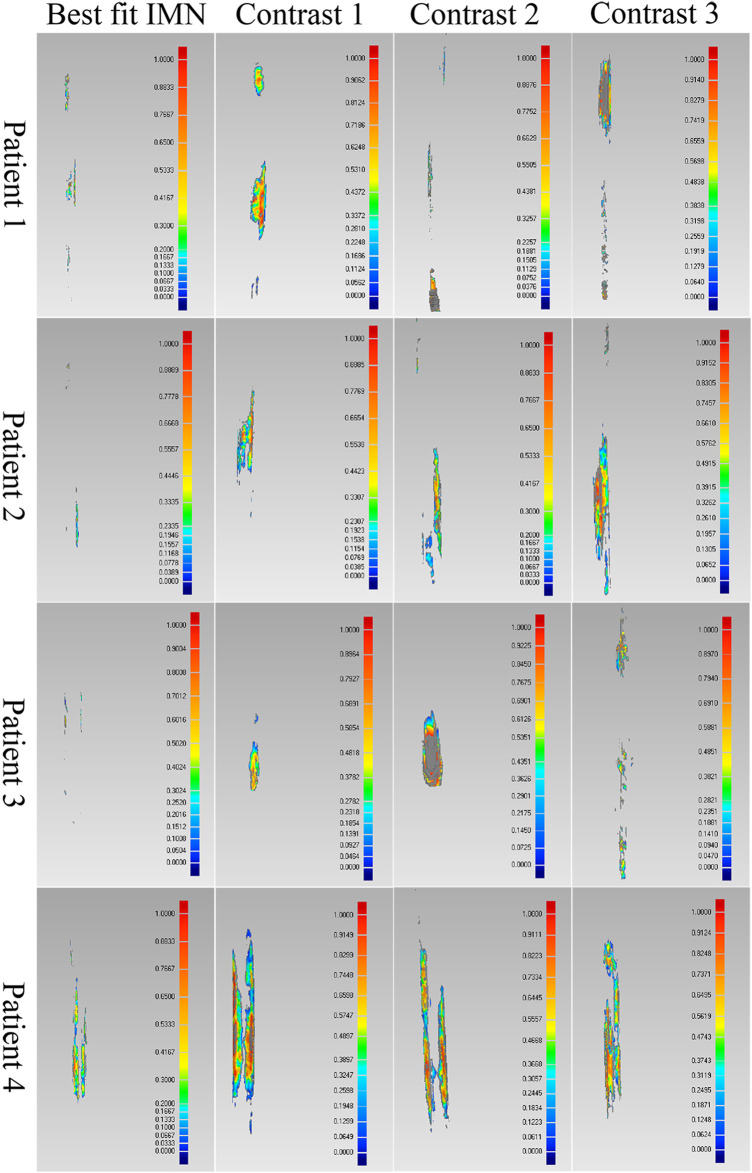
Comparison results of the optimal intramedullary nails for four patients. Different colors represent different wall thickness, the thickness increases gradually from blue to red, the maximum thickness is 1 mm, and gray indicates that the wall thickness here exceeds 1 mm.

**FIGURE 9:**
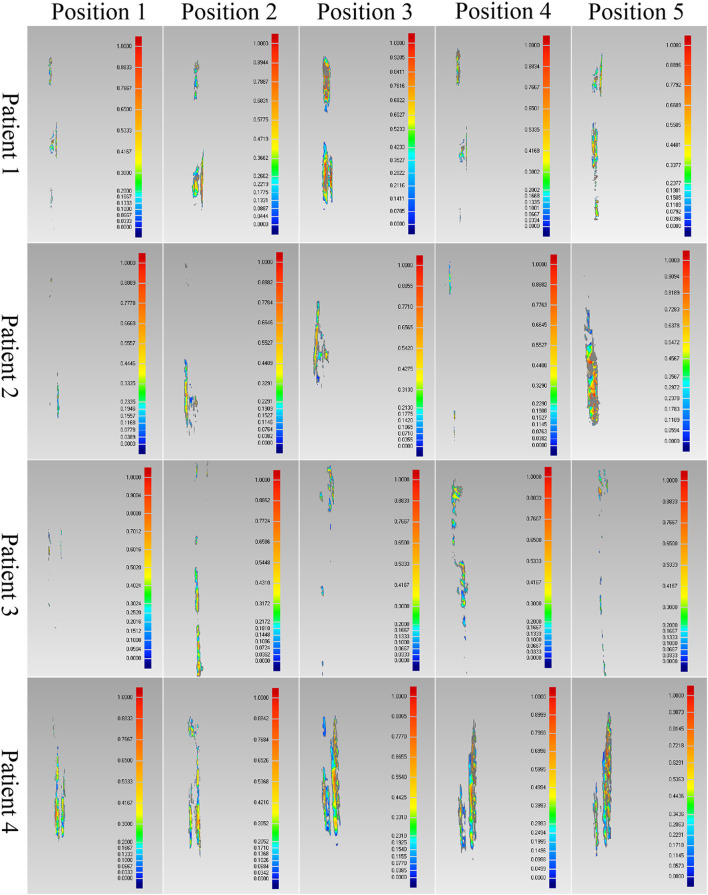
Comparison results of the optimal positions of four patients.

The proposed method was verified furtherly by comparing with a mature software (Geomagic Control 2014). According to the calculation of the proposed method, the most suitable IMNs of the four patients are the No.2, No.3, No.1 and No.3 IMNs successively. Patient 1 was taken as an example. The No.1, 2, 3 and 4 IMNs met the length and diameter requirements of patient 1. Among them, the No.2 IMN is determined by the proposed method, and the other nails were selected as a control group named contrast 1, 2 and 3. The femur model of patient 1 and the No.2 IMN model after transformation were imported into the software. The Boolean operation was employed to obtain an intersection from the surface of the MC model to the surface of the IMN model. The volume and wall thickness of the intersecting were calculated and recorded. The other three IMNs under their corresponding best transformation and the femur of patient 1 were imported into the software respectively. The volume and wall thickness of the intersecting parts were calculated. The remaining 3 patients performed the same operations. The numbers of the outside points of IMNs calculated by the proposed method were compared with the intersection volume in Figure 7A. The wall thickness distribution of the intersecting parts was shown in Figure 8. Next, the best registration position was verified. Patient 1 and its most suitable IMN were taken as an example. The best registration position obtained by the proposed method was recorded as position 1. The other four positions are selected: An optimal position calculated by the isthmus initial length which is different from the optimal growth scheme was arbitrarily selected as position 2. An optimal position different from the growth direction and growth speed of the optimal growth scheme was arbitrarily selected as position 3, and any two positions different from the best position under the optimal growth scheme were selected as position 4 and position 5. After being transferred to the five positions in turn, the IMN model and the femur model were imported into the software. The volume and wall thickness of the intersecting parts were calculated and recorded respectively. The remaining 3 patients performed the same operations. The numbers of the outside points of IMNs in five registration positions were compared with the intersection volume calculated by the analysis software in Figure 7B. The wall thickness distribution of the intersecting parts was shown in Figure 9.

As shown in the result, the volume and wall thickness of intersecting part of the IMN determined by the proposed method are smaller than those of the other three IMNs in the control group. The number of outside points calculated by the proposed method is proportional to the intersection volume. The same result is obtained for the verification of the best registration position. The volume and wall thickness of intersecting part in the best registration position are smaller than those in the other four positions. For some IMNs, it is found that the number of outside points is zero while the intersection volume is not zero. This is because the MC is not a regular cylindrical tube, and a slight error occurs in the operation of the layered synthesis of circles. However, the wall thickness caused by the error is still small. The average intersection wall thickness of the four patients under the best registration is 0.191 mm. This accuracy satisfies the clinical requirements.

### 3.3 printed model experiments

#### 3.3.1 Matching experiments of IMNs

**FIGURE 10:**
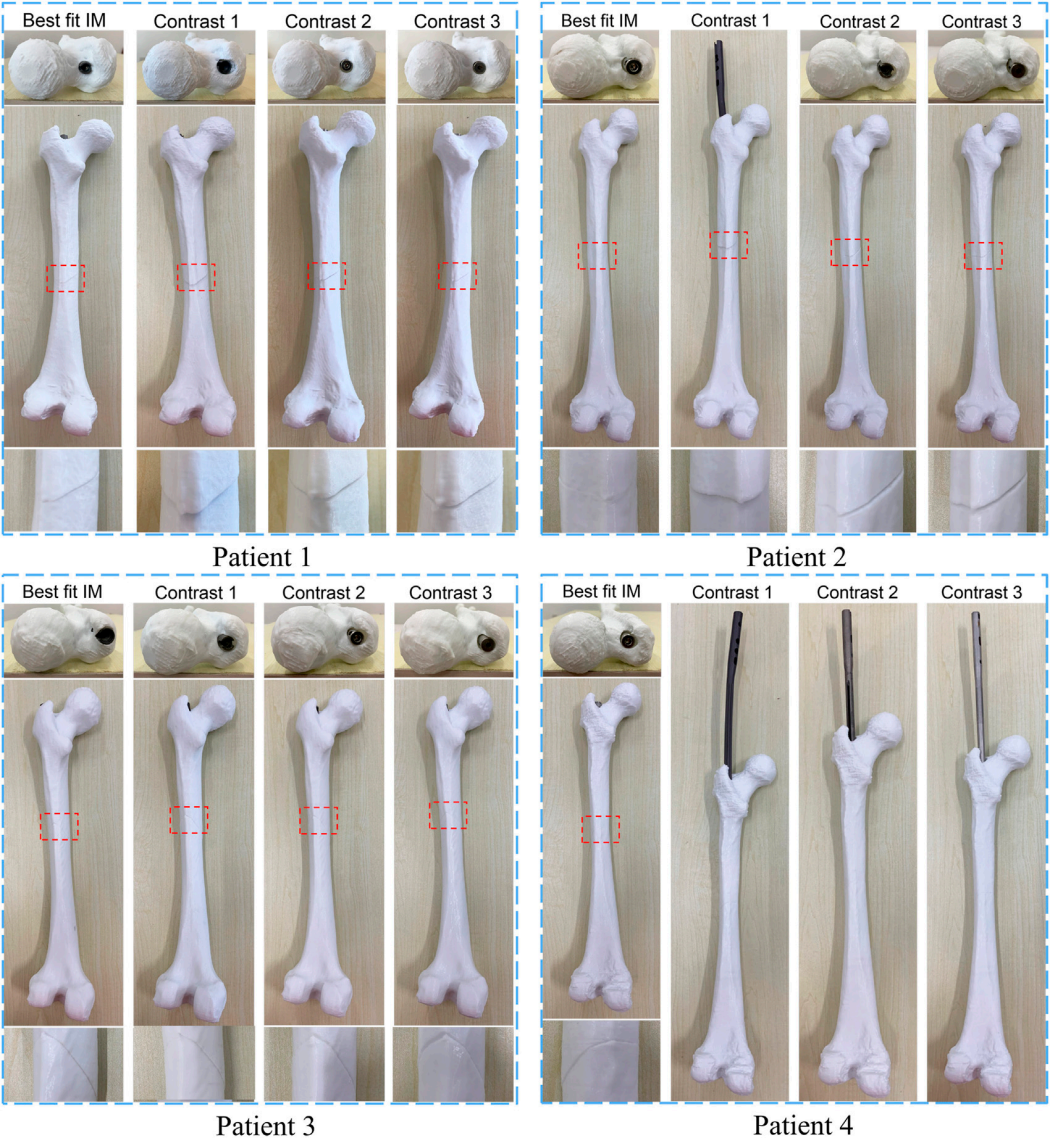
The result of matching experiments of intramedullary nails. The upper layer is the top view, and the alignment of the entry point and the intramedullary nail head is observed. The middle layer is a sagittal view, and the lower layer is a magnified view of the crack.

Whether the determined IMN can be successfully inserted into the patient’s MC was verified in this experiment. Instead of patients’ femurs, the 3D printed models of femurs were employed. The No.1, 2, 3 and 4 IMN met the length and diameter requirements of the femur of patient 1. Among them, the No. Two IMN was determined by the proposed method, and the other three were selected as a control group named contrast 1, 2 and 3. Four identical femur models of patient 1 were printed. To simulate broken bones, the four models were divided into two parts in the same section. The ICP was employed to register the femur model and its 3D image and get a transformation matrix *P*. Based on the transformation matrix, the entry point on the femoral model was obtained by transforming its position on the image. The four IMNs were inserted into femur models through their corresponding entry point. The same operations were performed for patient 2, 3 and 4. The alignments at the seams are shown in Figure 10.

The results showed that the broken bones of patient 1 fitted tightly with its optimal IMN. However, displacement or torsion occurred after the insertion of IMNs in the control group. The femur model of patient 2 matched well with its corresponding optimal IMN. In the control group, one IMN failed to be implanted. Because the curvature of the other two IMNs in the control group was less than that of patient 2 MC, the cracks were large. The femur model of patient 3 not only matched well with the optimal IMN but also matched well with the contrast 1 and contrast 2 IMNs. The optimal IMN was selected because of its larger diameter. The femur model of patient 4 matched well with its corresponding optimal IMN. However, the implantation of three IMNs in control group failed. When the three unsuitable IMNs were registered with the MC of patient 4, it was found that the outside points appeared on the left and right sides of the upper femur at the same time. The special distribution of outside points caused the failure. In short, compared with the IMNs in control group, the matching of the femur models and their corresponding optimal IMN determined by the proposed method was the best.

The proposed method can select the most suitable IMN for the target MC from a given number of IMNs, however, there is a possibility that the selected IMN may not be successfully inserted into the MC. We solve this problem by seeking the suitability threshold. The values of suitability *Φ* of all IMNs that meet the length and diameter requirements for 4 patients were calculated (Table 2).

**TABLE 2:** Suitability of different intramedullary nails for 4 patients.

		Best fit IM nail	Contrast 1	Contrast 2	Contrast 3
Suitability	Patient 1	0.121	0.353	0.195	0.577
	Patient 2	0.071	0.164	0.144	0.393
	Patient 3	0.081	0.096	0.095	0.216
	Patient 4	0.089	0.353	0.173	0.321

In the cases of model experiments, the maximal suitability of adaptation between IMN and MC is 0.121, while the minimal suitability of non-adaptation is 0.144. We take the mean of the two values as the suitability threshold. The value of the suitability threshold is 0.133. As shown in Table 2, the suitability of the optimal IMNs for the four patients is less than 0.133. Meanwhile, the femur models of four patients are well matched with their corresponding optimal IMNs in the model experiments. In addition, the suitability of the contrast 1 and contrast 2 IMNs for patient 3 are also less than 0.133, and the two IMNs are well-matched with patient 3. The suitability value of contrast 2 IMN for patient 2 is 0.144, and the crack is large after insertion of the IMN. The result indicated that an IMN with suitability less than 0.133 is available. Otherwise, the IMN is unavailable.

#### 3.3.2 Entry point valuation experiment

**FIGURE 11:**
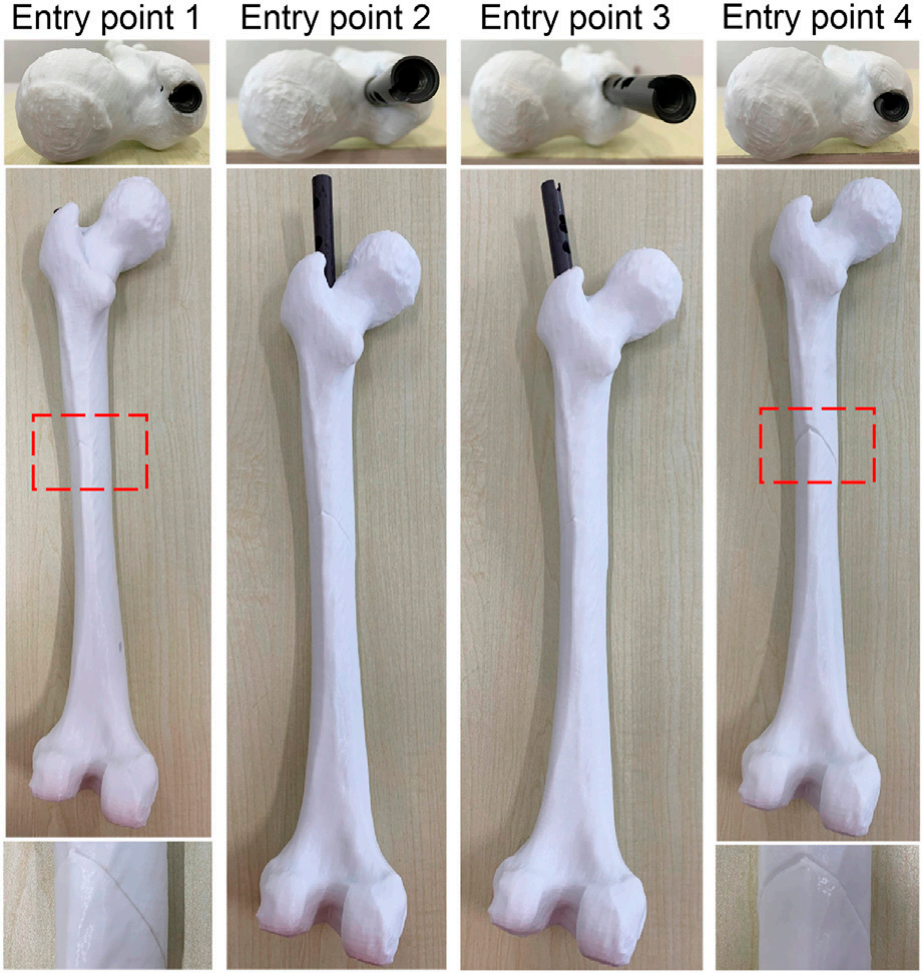
The result of entry point valuation experiments.

Each IMN has a suitable insertion position. An inaccurate position of the entry point may lead to implantation failure or deformation of an IMN. Surgeons usually reduce the diameter of the IMN to solve the problem. However, a smaller diameter leads to a decline in support strength. Therefore, an accurate location of the entry point is necessary. The experiment was taken on the femoral model of patient 3 and its most suitable IMN. The entry point determined by the proposed method was recorded as entry point 1. A point in piriform fossa was selected as entry point 2. Another two entry points were selected as entry point 3 and 4 according to the method in Farhang et al. (2014). Entry point 2 to 4 were selected as the control group. Four identical femur models of patient 3 were printed and drilled according to the positions of four entry points. The IMN was inserted through the four entry points respectively (Figure 11).

The IMN could successfully insert into MC through entry point 1 while the seam was well aligned. However, the IMN caused failed insertion through entry points 2 and 3. The IMN was successfully inserted through entry point 4, but the crack in the femur model was large. The results indicate that an appropriate entry point could greatly reduce the probability of implantation failure and deformation. The experiment verifies that the entry point determined by the proposed method is appropriate.

The time consumed by the proposed method was calculated in this experiment. All the steps were implemented on the standard PC (Intel Core i7-10870H 2.20 GHz, 16 GB memory). The average time of determining the most suitable one from four IMNs meeting length and diameter requirements for a patient is 236.8 s. This processing can be performed preoperatively. Therefore, the processing time meets clinical needs.

#### 3.3.3 Distal locking experiment

**FIGURE 12:**
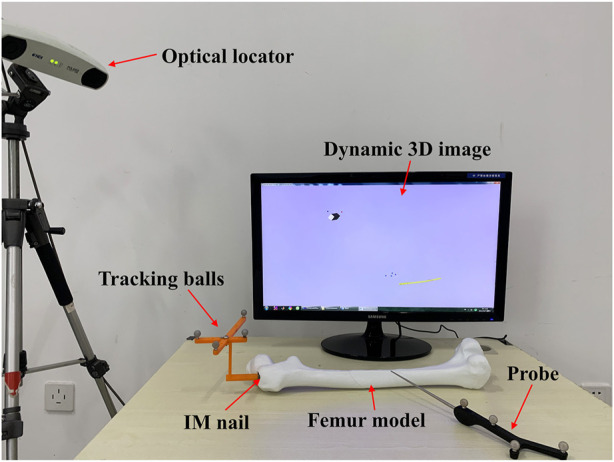
Composition diagram of distal locking system.

**FIGURE 13:**
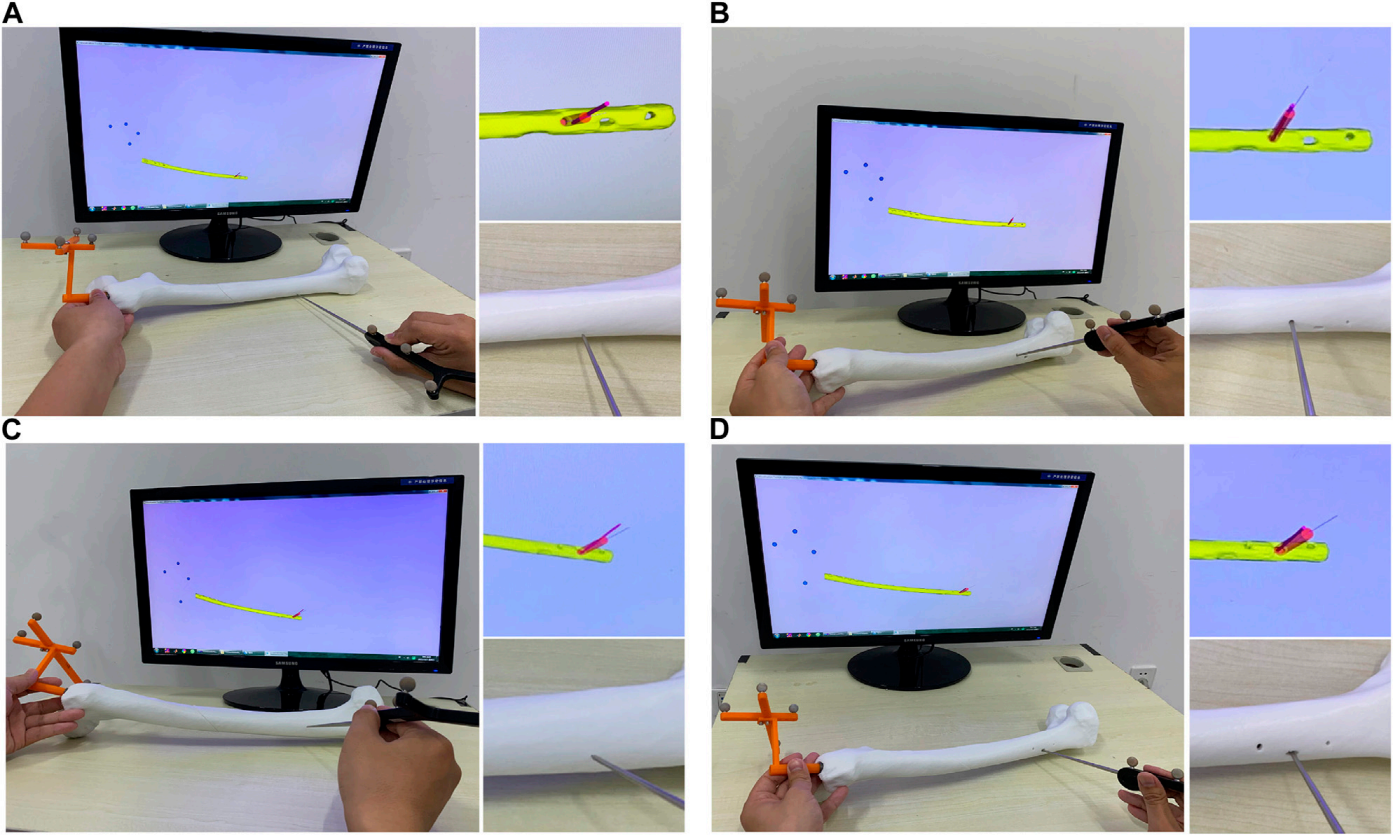
Experimental results of positioning of two distal holes. **(A)** Positioning of the distal hole 1. **(B)** Drilling of the distal hole 1. **(C)** Positioning of the distal hole 2. **(D)** Drilling of the distal hole 2.

The aim of this experiment is to verify whether the optimal IMN will deform after implantation. As we all know, most failure of distal locking guided by extracorporeal aimers is due to the displacement or torsion of IMNs. Surgeons usually locate distal holes through multiple X-ray imaging, which will pose health risks. We designed a three-dimensional image navigation system (Figure 12) for distal locking. The experiment was taken on the femoral model of patient 3 and its most suitable IMN. The positions of the IMN 3D image and bone drill 3D image were displayed in the guide window in real time. The IMN image could follow the movement of IMN entity in the window. Even if the IMN was implanted into the MC with no sight, the precise positions of distal holes could be located by observing the movement of the IMN image in the window. The processing flow is as follows: Firstly, a rigid body with four spheres was fixed at the IMN head (Figure 1H). The ICP was employed to register the IMN entity and its 3D image. The spatial coordinate of IMN image was mapped to the coordinate system of the position sensor. Secondly, VTK was employed to create a visual guide window. Another rigid body with four spheres was equipped on a bone drill. The bone drill was replaced by a probe in this experiment. Thirdly, distance and angle conditions were installed for distal hole positioning. The distance between the probe tip and the central axis of a distal hole should be less than 2 mm. The angle between the probe central axis and the central axis of a distal hole should be less than 15°. Finally, a red cylinder would appear on the central axis of the distal hole when the probe tip reached the requirements. Two distal holes of the IMN were located in this experiment. The obtained positions were marked and drilled on the surface of the femoral model. The probe could successfully pass through the distal holes as shown in Figure 13.

The two distal holes are accurately located within 28.54 s. The result indicates that the shape of IMN after implantation is consistent with its 3D image obtained in pre-operation. Therefore, the IMN determined by the method is not deformed.

## 4 Discussion

Both an unsuitable IMN and inaccurate positioning of the entry point will lead to deformation of an IMN after implantation. Various researchers have reported their solutions from the perspectives of measuring MCs shape (Buford et al., 2014; Liaw et al., 2019), MCs classification (Chen et al., 2018), and predicting the offset of distal holes (Mortazavi et al., 2019). These studies provide important guidance for the design and selection of IMNs. However, the first two methods do not take into account the influence of entry points, and the last method does not think about the optimization problem. A method to determine the optimal IMN with an entry point based on centerline adaptive registration is proposed in this study, which fundamentally eliminates the possibility of IMN deformation. The IMN determined by the proposed method fits tightly with the MC without squeeze. The IMN is implanted smoothly without reaming or hammering, which does smaller damage to the intramedullary blood supply. The proposed method can determine the largest diameter IMN with as little damage to the intramedullary tissue as possible. However, the robustness of the nail support strength needs to be further measured. We plan to establish a multibody model (Putame et al., 2020) of the IMN for verification in future work.

de Almeida et al. (2017) developed an automated workflow to extract the femoral neck axis, the femoral middle diaphysis axis, both trochanters, and the center of the femoral head. According to the extracted parameters, the surgeon can determine which prosthesis design and size to use in every patient-specific coupling. This method provides a very effective preoperative guide for total hip arthroplasty. But the determination of prosthesis depends on the personal experience of surgeons. Our method can calculate the suitability of an IMN for a specific patient by measuring the geometric quantities reflecting the interference between the femur and nail, and the suitability values of all nails are compared to determine the most suitable one automatically. In addition, de Almeida et al. also indicated that the proposed toolbox does not take into account the femur length. But the length and diameter of femur are used as important indicators in our method.

The method is verified by the femur models of 4 patients. The experiment results show that when the IMN is registered with the MC in the optimal position, the number of outside points is less than any other registration position. The number of outside points of the determined IMN is less than any other IMN in the control group. The registration effect of the determined IMN is also confirmed by the geometric analysis software.

In clinical surgery, the deformation of IMNs often leads to the failure of distal locking guided by an extracorporeal aimer. Surgeons usually collect X-ray images of the distal end of IMN with the assistance of C-Arm. Some studies say that as many as 48 X-ray images are needed to successfully fix the screw (Diotte et al., 2015). The imaging increases the operation time greatly and causes radiation damage to the medical staff and patients. In order to develop a distal hole location system with high positioning accuracy, intuitive guidance mode and no radiation during surgery, many studies have been carried out. For example, Choi et al. (2017) proposed a new laser-guided navigation system. The system has a handle-integrated line laser marking and inertial and magnetic sensor unit. The drill insertion point can be targeted onto skin and be perpendicular to the center of the distal hole. Liao et al. (2010) developed a surgical navigation system. The system combines a laser guidance technique with a three-dimensional (3D) autostereoscopic image overlay technique. The average error of distal holes positioning is 2.48 mm. The calibration method of this system is based on the transformation matrix between the affected bone and its 3D image, so no displacement and deformation of the affected bone after implantation is an important guarantee of the system’s accuracy. The above two systems have the characteristics of non-radiation, simple operation and intuitive guidance, but both are based on no deformation. Choi et al. (2017) also indicated that an appropriate compensation technique must be introduced for the deformation of IMNs. Our method proposed in this study can effectively solve the deformation of IMNs. Therefore, our method can be used as a preparation for optical navigation systems and extracorporeal aimers.

A 3D image navigation system is designed in the distal locking experiment. The linkage is realized by registering the IMN entity and its 3D image. The spatial position of the IMN is displayed in real time, and the accurate positions of distal holes are located under the guidance of optical navigation. The system has the characteristics of user-friendly, intuitive guidance, simple operation and radiation-free during surgery.

A problem occurs in the model experiments. Even for the optimal IMN, there may be some obstacles during insertion. Rotations and swing can ease the insertion. The problem should be very common in clinic. Therefore, our future work will focus on the path planning of IMN insertion.

## 5 Conclusion

An inappropriate IMN or inaccurate positioning of an entry point will lead to deformation during IMN insertion. The deformation is the main reason for the difficulty of distal locking. A method based on centerline adaptive registration is proposed to solve the problem. The method can determine the optimal IMN and an entry point. The method solves the deformation fundamentally and can determine the largest diameter IMN with as little damage to the intramedullary tissue as possible. The effectiveness of this method is proved by experiments. The results show that the determined IMN can be successfully implanted into the MC and the broken bones can be restored well. On the whole, we fixed suitability parameter limits as a pre-screening tool to determine nails which can be successfully used. Minimally invasive, accurate, intelligent and personalized surgery is the inevitable trend of orthopedic treatment in the future. The method proposed in this study follows the trend closely. Next, we will further verify its clinical effectiveness and make it reach the level of clinical application with the help of surgeons.

## Data Availability

The original contribution presented in the study are included in the article/supplementary material, further inquiries can be directed to the corresponding author.
